# Supplementary figures and images for: Dual genome-wide CRISPR knockout and CRISPR activation screens identify mechanisms that regulate the resistance to multiple ATR inhibitors
Source: PLoS Genet. 2020 Nov 2;16(11):e1009176. doi: 10.1371/journal.pgen.1009176 (PMC7660927; doi:10.1371/journal.pgen.1009176)

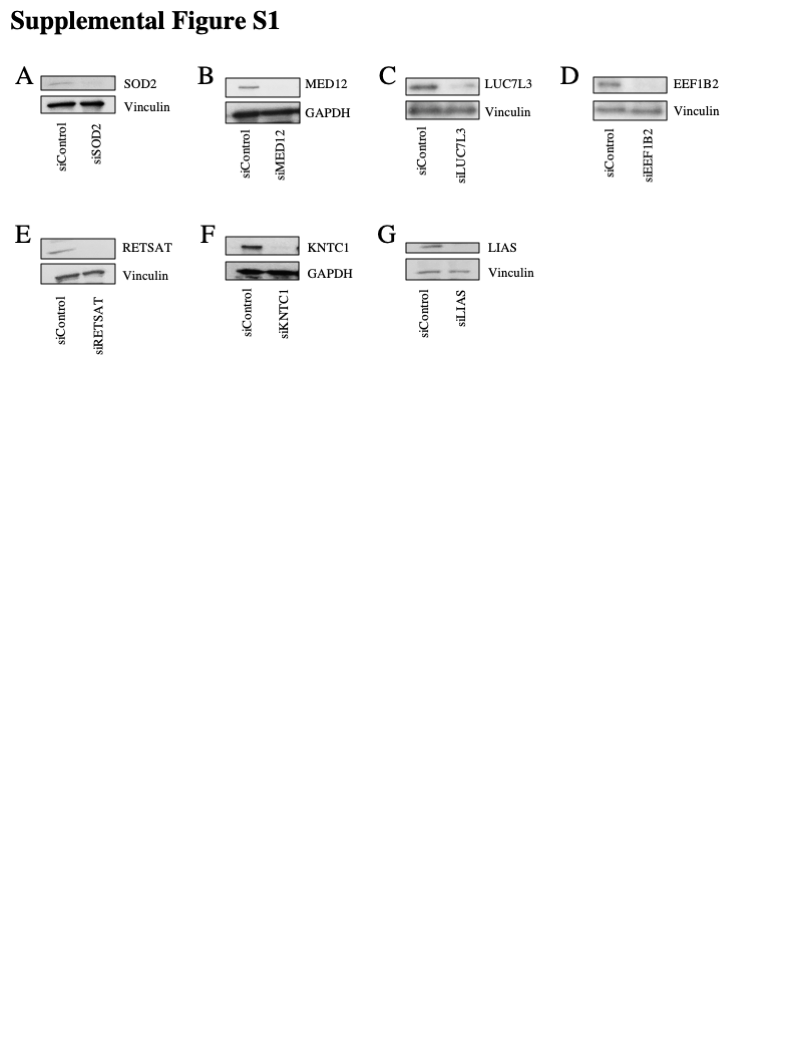

Supplement: S1 Fig — Western blots showing knockdown of SOD2 (A), MED12 (B), LUC7L3 (C), EEF1B2 (D), RETSAT (E), KNTC (F), and LIAS (G) in HeLa cells are presented. (TIF) [file pgen.1009176.s001.tif]

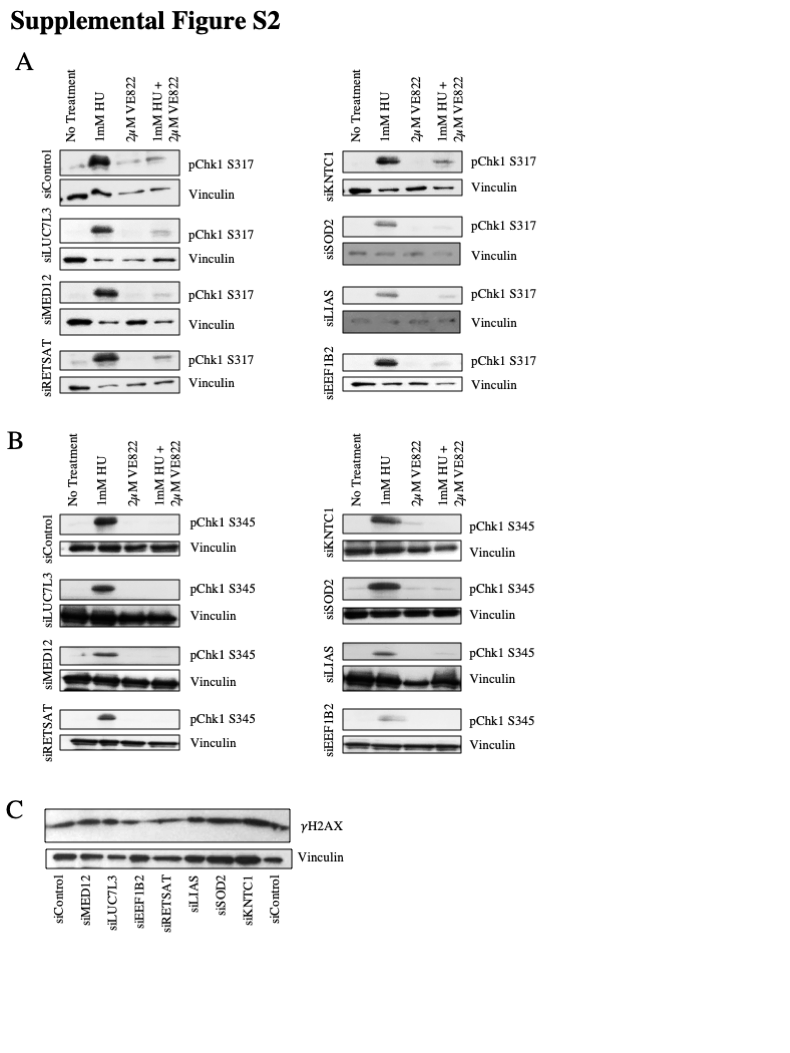

Supplement: S2 Fig — Western blots showing the levels of pCHK1 S317 (A) and pChk1 S345 (B) in HeLa cells after knockdown of the top hits followed by 24 hours of no treatment, hydroxyurea treatment, ATRi treatment, or hydroxyurea with ATRi treatment, are shown. (C) Western blots showing no impact on γH2AX levels upon knockdown of the top hits in HeLa cells. (TIF) [file pgen.1009176.s002.tif]

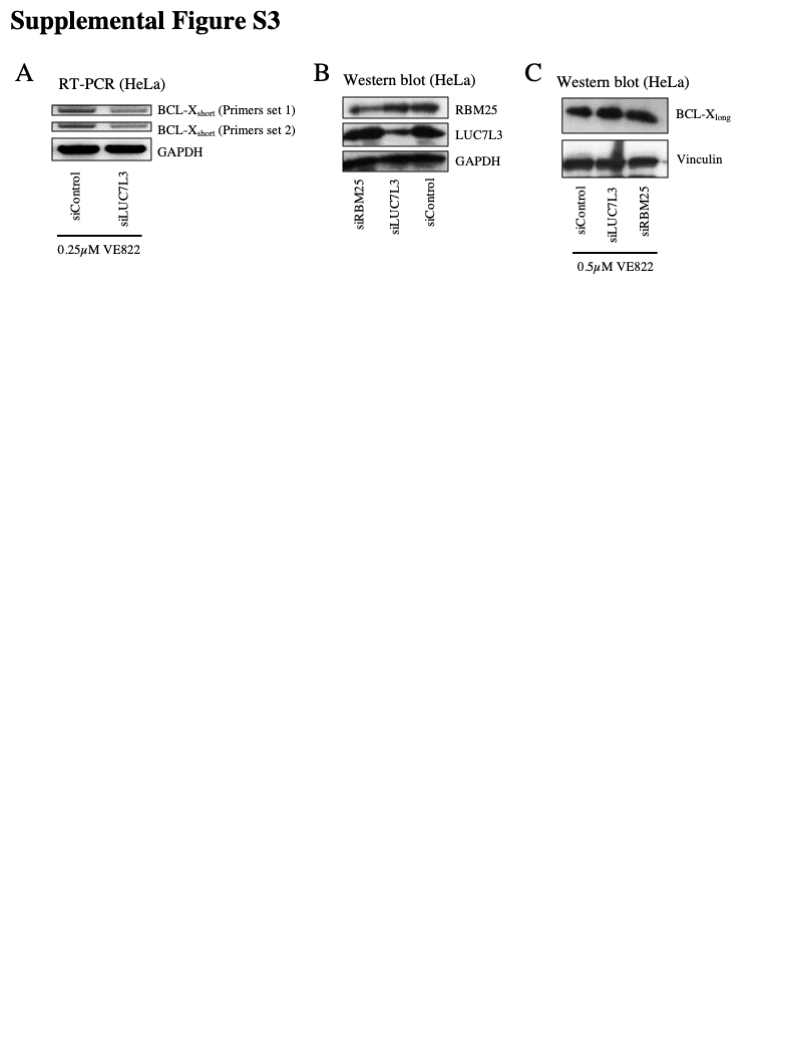

Supplement: S3 Fig — (A) Reverse transcriptase PCR showing a decrease in BCL-Xshort mRNA after knockdown of LUC7L3 followed by ATRi treatment in HeLa cells. Two different primer sets for BCL-Xshort were used, and GAPDH was used as control. (B) Western blots showing RBM25 depletion by siRNA-mediated knockdown in HeLa cells. (C) Western blot showing an increase in the protein levels of the BCL-Xlong isoform in HeLa cells after knockdown of LUC7L3 or RBM25 and ATRi treatment. (TIF) [file pgen.1009176.s003.tif]

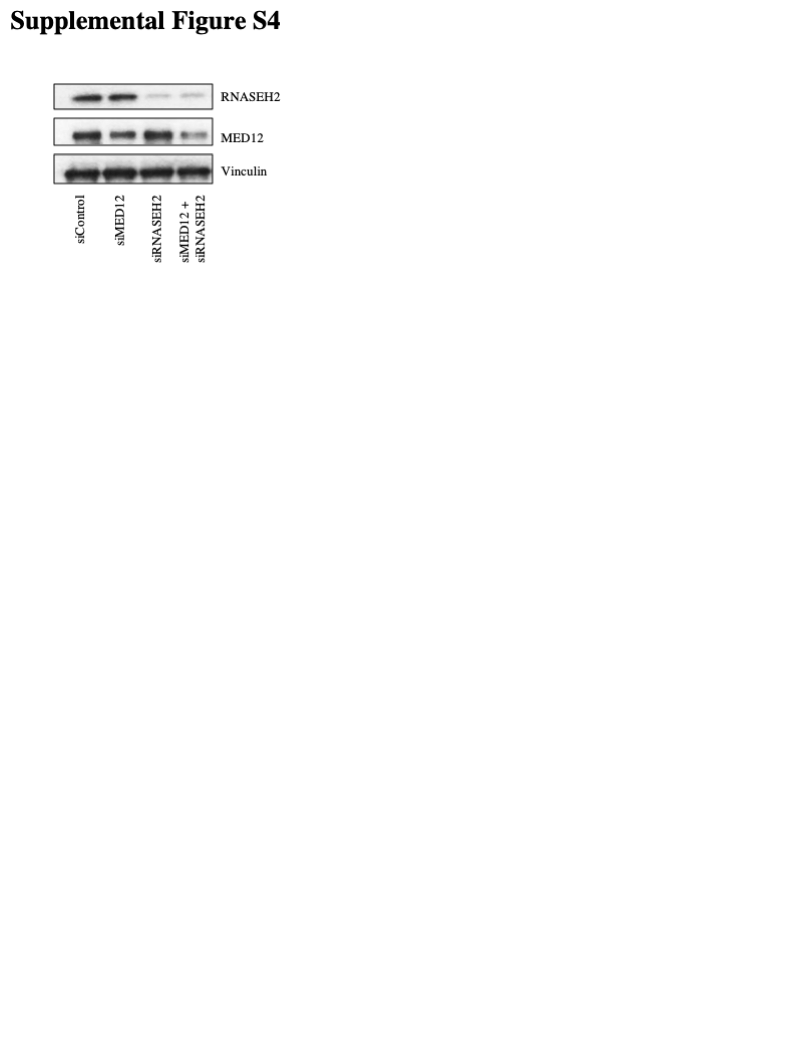

Supplement: S4 Fig — Western blots showing the siRNA-mediated co-depletion of RNASEH2 and MED12 in HeLa cells are presented. (TIF) [file pgen.1009176.s004.tif]

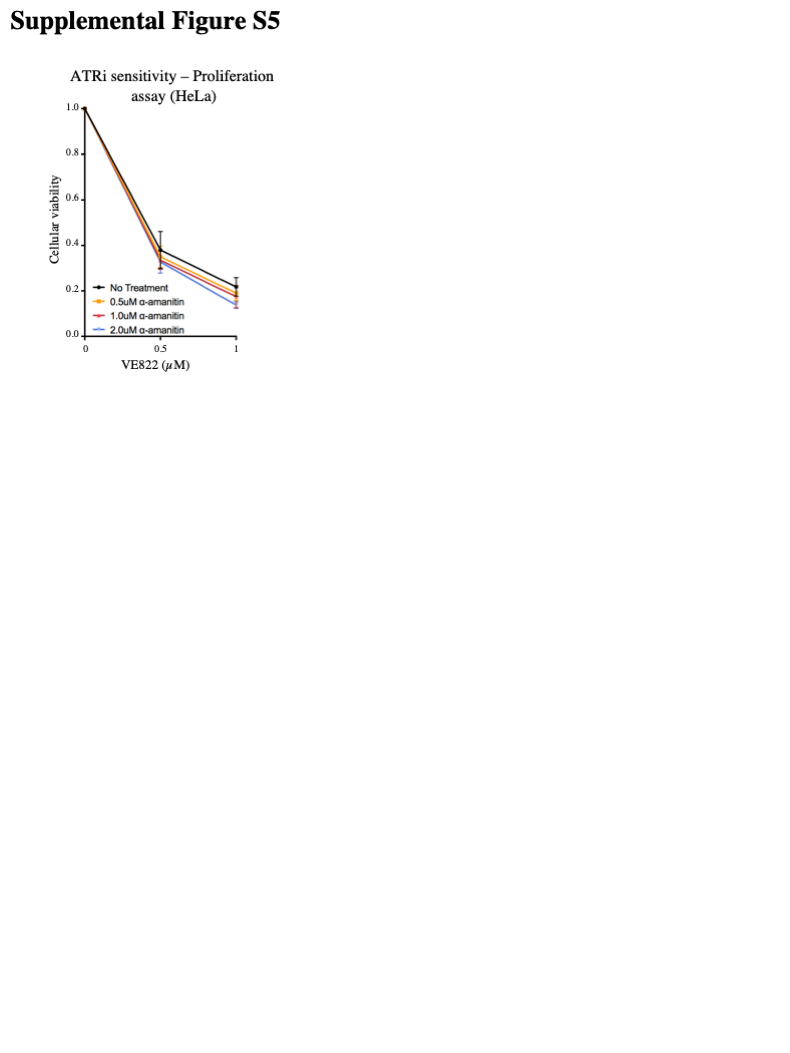

Supplement: S5 Fig — Cellular proliferation experiment showing that inhibition of RNA polymerase II using α-amanitin does not impact VE822 sensitivity of HeLa cells. Cells were treated with both drugs for 3 days at the indicated concentrations. The average of four experiments is shown, with error bars representing standard deviations. (TIF) [file pgen.1009176.s005.tif]

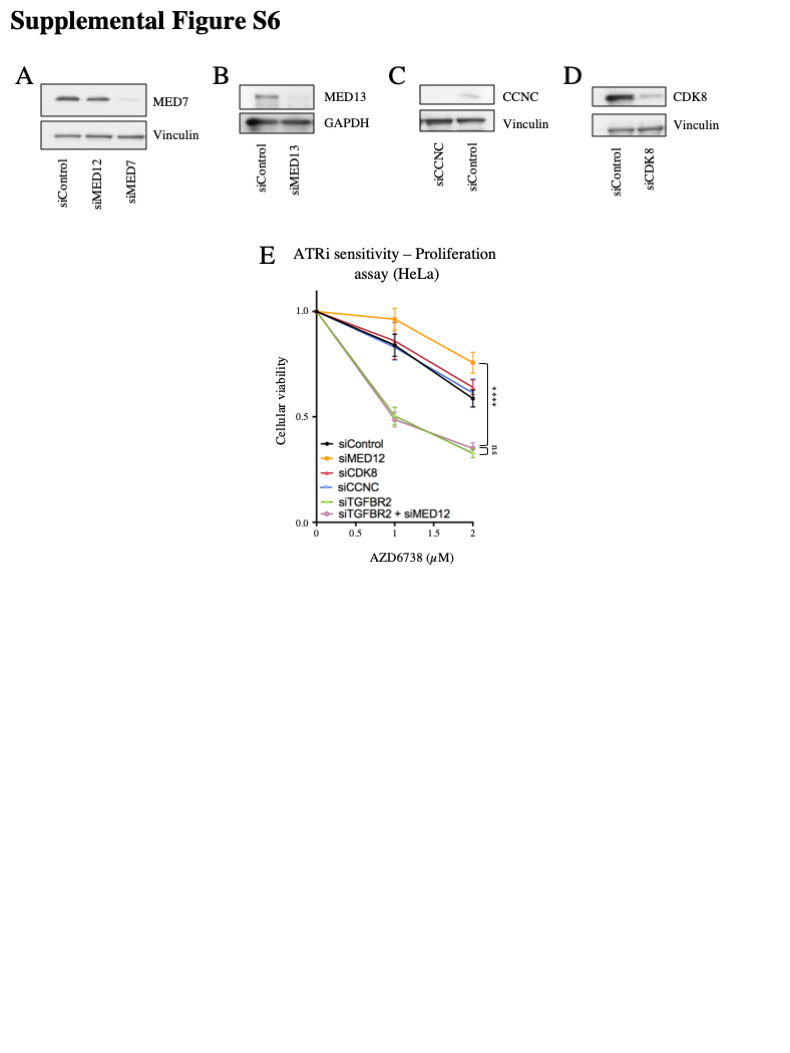

Supplement: S6 Fig — (A-D) Western blots showing knockdown of MED7 (A), MED13 (B), CCNC (C), and CDK8 (D) in HeLa cells are presented. (E) Cellular proliferation experiment showing the AZD6738 sensitivity of HeLa cells upon depletion of MED12, CDK8. CCNC, or TGFBR2. The average of three experiments is shown, with error bars representing standard deviations. Asterisks indicate statistical significance. (TIF) [file pgen.1009176.s006.tif]

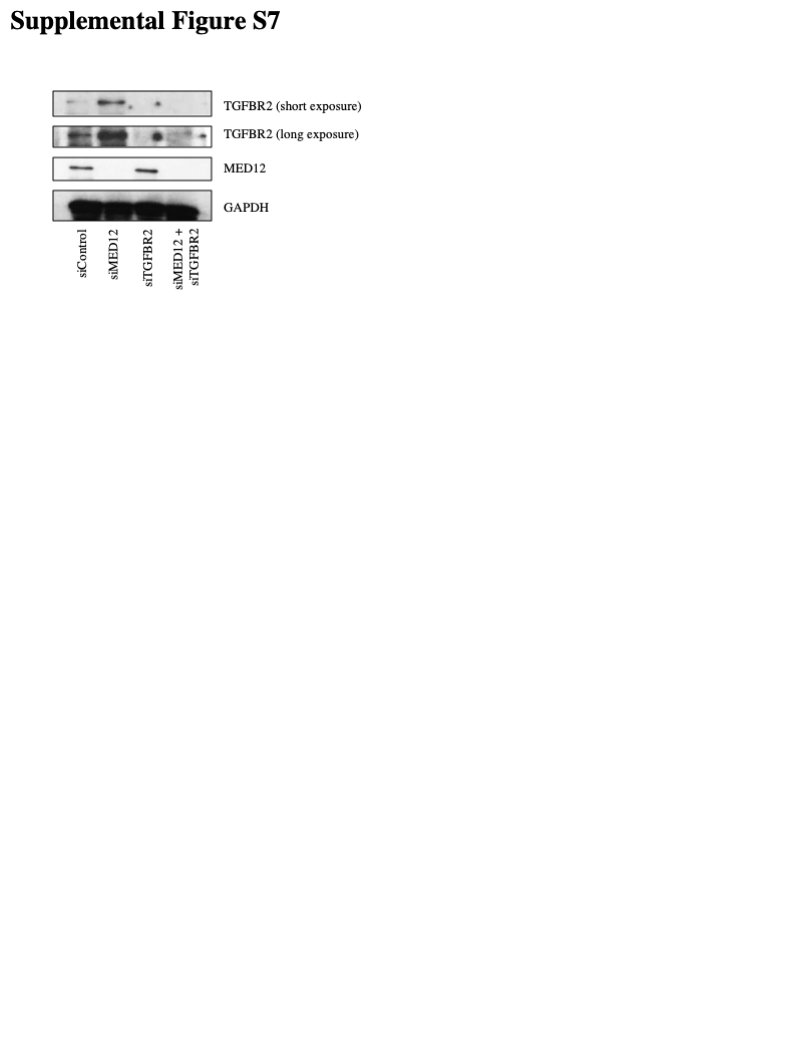

Supplement: S7 Fig — Western blots showing the siRNA-mediated co-depletion of TGFBR2 and MED12 in HeLa cells are presented. (TIF) [file pgen.1009176.s007.tif]

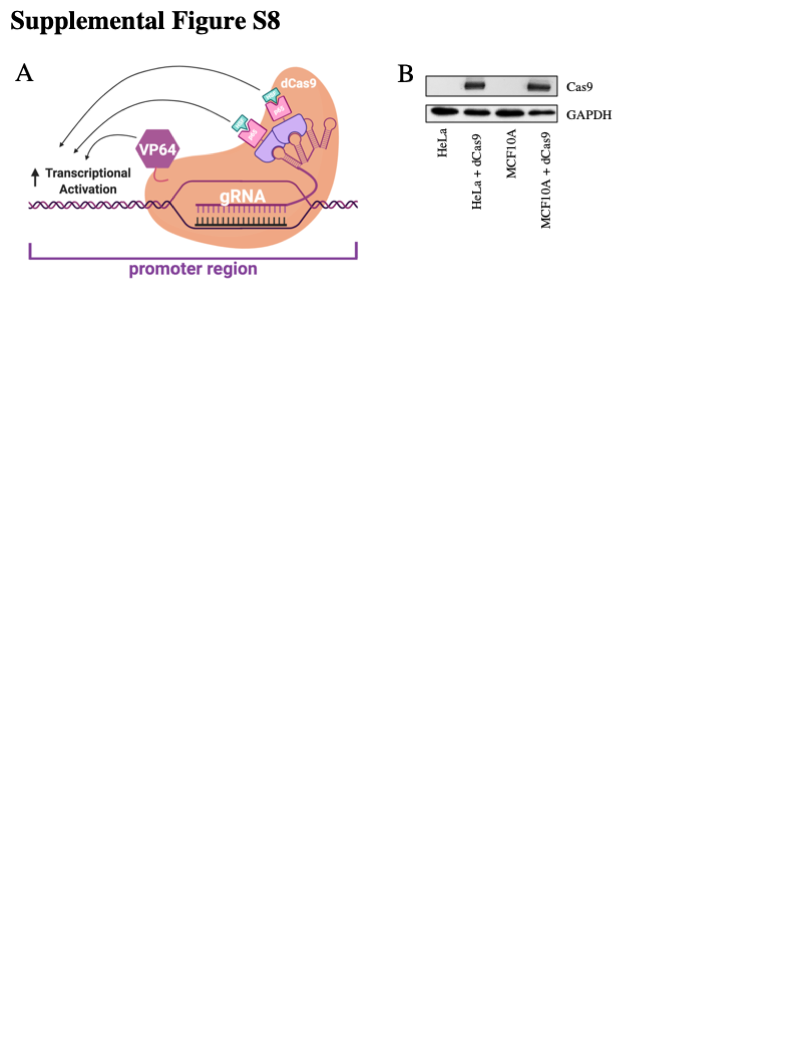

Supplement: S8 Fig — (A) Schematic representation of the CRISPR activation screen setup. The gRNA targets dCas9 to the promoter region of the gene of interest, along with multiple transcriptional activators to upregulate the transcription of the gene. (B) Western blots showing dCas9 expression in the cells used for the CRISPR activation screen and HeLa-dCas9 overexpression cell lines. (TIF) [file pgen.1009176.s008.tif]

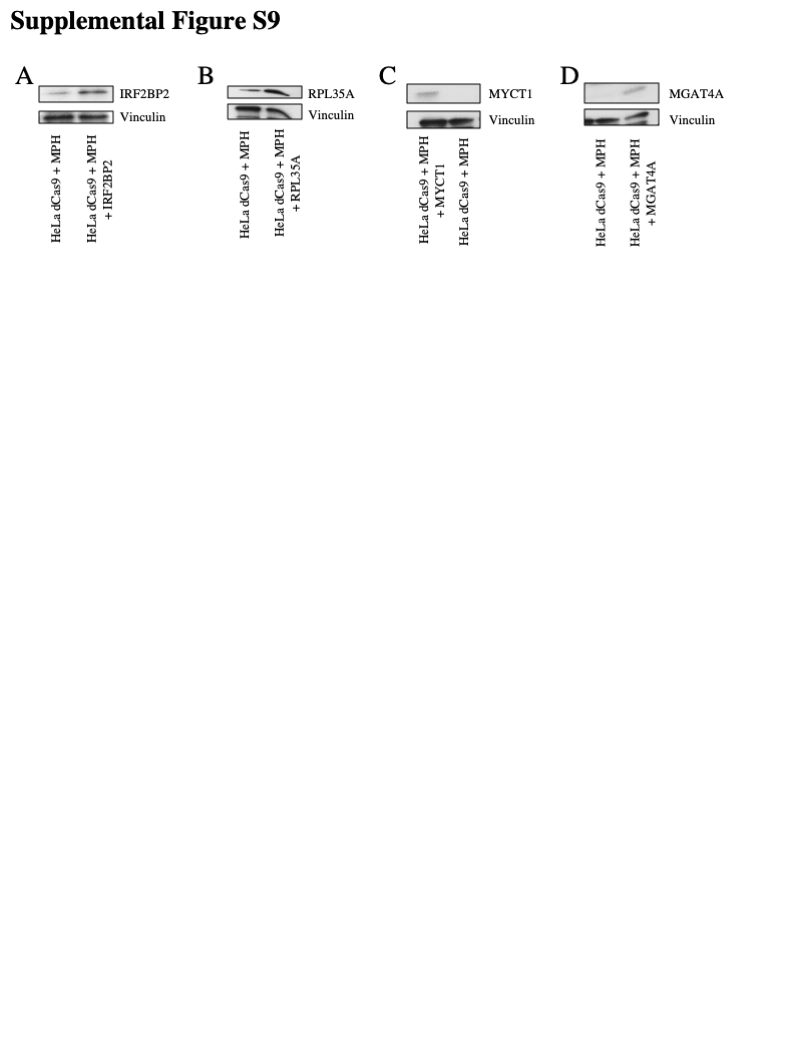

Supplement: S9 Fig — Western blots showing the overexpression of IRF2BP2 (A), RPL35A (B), MYCT1 (C) and MGAT4A (D) in HeLa cells are presented. (TIF) [file pgen.1009176.s009.tif]
