# Supplementary material for: Dual genome-wide CRISPR knockout and CRISPR activation screens identify mechanisms that regulate the resistance to multiple ATR inhibitors
Source: PLoS Genet. 2020 Nov 2;16(11):e1009176. doi: 10.1371/journal.pgen.1009176 (PMC7660927; doi:10.1371/journal.pgen.1009176)
Supplement: Attachment — Submitted filename: Response to referees.docx [file pgen.1009176.s016.docx]

**Response to referees**

We would like to thank the reviewers for their constructive comments, which led to a significantly improved manuscript. To address the reviewers’ concerns, we are submitting a substantially revised manuscript with 36 new figure panels, as well as 10 revised figure panels. Below, please find our point-by-point reply to reviewers’ comments (our responses in red font)

**Reviewer #1**

We are glad that the reviewer found that the data presented in our manuscript “*are solid and rigorous and will stimulate research in the area, and hence is an important advance”.* We thank the reviewer for their helpful comments. We have addressed these comments in the revised manuscript as indicated below:

*1. I request generating a summary table as a main Table (not supplemental) of all these 7 genes & all the effects tested, including the effects that are common among them (like a "-" for reactivates ATR). Include those phenotypes tested in multiple cell types. The findings in each cell of the table could either be quantitative with fold-effects or a qualitative, depending on what the authors determine is most rigorous/representative.*

We thank the reviewer for this useful suggestion. We included this table as the **new Fig 5D.**

*2. The title could be more focused on the broad conclusion of "distinct mechanisms mediate ATR-inhibitor resistance" or something, since I'm not seeing that the two types of screening found "common mechanisms."*

In the original title, by “common” we meant that the candidates investigated were found to mediate resistance to both ATR inhibitors studied, not that the mechanisms were common between the hits. We agree with the reviewer the original title is potentially misleading, and we omitted the word “common” in the title of the revised manuscript. The new title of the manuscript is: “***Dual genome-wide CRISPR knockout and CRISPR activation screens identify mechanisms that regulate the resistance to multiple ATR inhibitors”.***

*3. The figure labeling could have a bit more information. In figure panels themselves, I request that the authors state the name of the cell line. If the same cell line is used in all the panels, then naming it in the "A" panel would be sufficient. For panels, like 2A, it can be useful to state what we are looking at, like "Gene knockouts causing increased fitness in HeLa."*

We thank the reviewer for this suggestion. In the revised manuscript, we added titles to all figure panels and indicated the cell lines used in each figure panel

*4. Regarding interpretation, I think it is important to have a Discussion paragraph that describes the pros/cons of treating cells with a high-dose for a relatively short period of time. Namely, examining a higher dose over a shorter period of culturing may bias the screen towards pathways affecting apoptotic death, whereas a lower dose over a longer period of culturing may bias the screen towards pathways affecting chromosomal instability that causes death by mitotic catastrophe. This is a recurrent theme with PARP inhibitors, and so that literature may help with the paragraph, like this paper PMC5791853, but I am not specifically requesting the citation, this is just an example of the literature I'm talking about. The clonogenic survival validation assays are helpful in this extent (i.e. the top genes affect both acute sensitivity and long-term sensitivity), but this should be discussed.*

We thank the reviewer for this thoughtful comment. We have included a paragraph in the Discussion section of the revised manuscript addressing this point.

**Reviewer #2**

We are glad that the reviewer found that our manuscript contains “*three main novel angles”* and addresses an *“important but currently understudied aspect of cancer therapy”.* However, the reviewer indicates that we *“do not provide sufficient insight into the actual mechanisms behind tumour resistance to ATR inhibition upon loss of these genes”.* In their Specific comments, the reviewer suggests experiments to improve the mechanistic aspects of our manuscript, related to LUC7L3 and MED12.

We thank the reviewer for these very helpful suggestions, which allowed us to markedly improve our manuscript. In the revised manuscript, we present significant mechanistic insights for both factors. In particular, we show that the role of MED12 in ATRi sensitivity is independent of the Mediator complex or of transcription in general. Instead, we show that MED12 suppresses the TGFβ signaling pathway and its loss promotes ATRi resistance and increased replication fork speed by hyper-activating this pathway through increased expression of the TGFβ Receptor 2. We also show that LUC7L3 mediates the ATRi response through BCL-X alternative splicing in conjunction with its partner RBM25. These findings are described below in the response to the reviewers’ Specific comments. We sincerely hope that the reviewer will find the revised manuscript describing these mechanistic insights to be satisfactory for publication.

*Specific comments:
1. For the top seven hits identified in CRISPR KO screens, the common mechanism of ATRi resistance is reduction in apoptosis (Fig. 4B). However, only one of these genes, LUC7L3, appears to have a possible direct link to controlling apoptosis. As LUC7L3 had previously been shown to interact with RBM25 and the latter controls expression of the pro-apoptotic form of BCL-X, the authors tested if loss of LUC7L3 would result in decrease of pro-apoptotic BCL-X and they saw exactly that (Fig. 4D). But what is the molecular mechanism of LUC7L3 in RBM25/BCL-X control? E.g., does LUC7L3 affect stability and/or expression levels of RBM25? Does LUC7L3 re-expression in depleted cells complement for the loss of the protein? Regarding the rest of the hits affecting apoptosis, analysing transcriptomes of the depleted cells could have shed light on the changes in gene expression and provided possible links to regulation of cell fate decisions.*

A molecular mechanism for how the LUC7L3-RBM25 interaction affects BCL-X splicing was previously proposed (PMID: 18663000). We apologize if this was not clearly explained in our original manuscript. LUC7L3 was originally identified as a component of the U1 snRNP complex which affects 5′ splice site selection in yeast and human cells (PMID: 17726058). Subsequently, Zhou et al (PMID: 18663000) identified RBM25 as a splicing factor which binds to the exonic splicing enhancer sequence CGGGCA in the BCL-X pre-mRNA, and facilitates the alternative splicing of the BCL-X_short_ isoform. Moreover, they found that RBM25 interacts with LUC7L3. While they never tested if LUC7L3 depletion affects BCL-X splicing, they proposed that RBM25 recruits the U1 snRNP complex through its interaction with LUC7L3, to initiate this alternative splicing. Since the impact of LUC7L3 on BCL-X splicing was never directly tested, we thought it was important to address this, and we present the first (to our knowledge) direct demonstration that LUC7L3 is involved in BCL-X splicing, thus validating the previously proposed model. We now clearly explain this in our revised manuscript.

Moreover, in the revised manuscript we extended our analyses to RBM25, to confirm the involvement of this mechanism in ATRi resistance. In fact, RBM25 scored highly in our original screens, ranking #201 in the AZD6738 screen. Indeed, in the revised manuscript, we show that RBM25 depletion results in ATR inhibitor resistance (**new Fig 4C-D**). Moreover, we show that similar to loss of LUC7L3, RBM25 depletion reduces the levels of the pro-apoptotic BCL-X_short_ isoform upon ATRi treatment (**new Fig 4F**). As requested by the reviewer, we also investigated the levels of RBM25 upon loss of LUC7L3 and found no significant change (**new Fig S3B**). Overall, these findings indicate that LUC7L3 exerts its effect on ATRi resistance through its partner RBM25 and indicate that the alternative splicing of the BCL-X apoptosis regulator by the LUC7L3-RBM25 pathway modulates the cellular resistance to ATR inhibitor treatment.

Unfortunately, we were not able to obtain a construct or some other system for re-expression of LUC7L3 in the time available to us for the revision. However, we would like to point out that the fact that we confirmed that loss of LUC7L3 results in ATRi resistance through both CRISPR-mediated knockout (in the original screen) and siRNA-mediate depletion (in the follow-up validation experiments) rules out that the phenotype observed is caused by some unspecific off-target effect.

We also performed RNA-sequencing experiments on select hits, as requested by the reviewer, but unfortunately this was not useful in identifying potential mechanisms. The pathways found to be differentially regulated were, in general, not common between the different hits, and did not point out to specific mechanisms. As an example, we present below the results of RNA-sequencing experiments performed in cells depleted of LUC7L3 or MED12, as well as MED7 as a negative control. The tables show the top 10 KEGG pathways found to be upregulated or downregulated upon knockdown of these factors, compared to control cells.

| **Top 10 KEGG pathways downregulated in LUC7L3-depleted HeLa cells treated with VE822** | **p-value**  **(n=3)** | **Top 10 KEGG pathways downregulated in LUC7L3-depleted HeLa cells treated with VE822** | **p-value (n=3)** |
| --- | --- | --- | --- |
| Carbon metabolism | 0.00036412 | Glycosaminoglycan biosynthesis - heparan sulfate / heparin | 0.00010485 |
| Glycolysis / Gluconeogenesis | 0.000463 | Glycosaminoglycan biosynthesis - chondroitin sulfate / dermatan sulfate | 0.00059836 |
| Epstein-Barr virus infection | 0.00106605 | Sphingolipid signaling pathway | 0.00067374 |
| AMPK signaling pathway | 0.00130996 | Amoebiasis | 0.00098787 |
| Kaposi sarcoma-associated herpesvirus infection | 0.00140381 | Rap1 signaling pathway | 0.00165045 |
| Homologous recombination | 0.00144661 | Parathyroid hormone synthesis, secretion and action | 0.00259044 |
| Cellular senescence | 0.00149433 | Pathways in cancer | 0.00288385 |
| Small cell lung cancer | 0.00424961 | cAMP signaling pathway | 0.00296414 |
| Spinocerebellar ataxia | 0.0046539 | Platelet activation | 0.00296498 |
| Fructose and mannose metabolism | 0.00521783 | Human papillomavirus infection | 0.00371105 |

| **Top 10 KEGG pathways downregulated in MED12-depleted HeLa cells treated with VE822** | **p-value**  **(n=3)** | **Top 10 KEGG pathways downregulated in MED12-depleted HeLa cells treated with VE822** | **p-value (n=3)** |
| --- | --- | --- | --- |
| Influenza A | 3.89E-08 | Ribosome | 2.29E-29 |
| Hepatitis C | 5.48E-08 | RNA transport | 5.74E-11 |
| Cytokine-cytokine receptor interaction | 4.18E-07 | Cell cycle | 2.00E-10 |
| NOD-like receptor signaling pathway | 6.26E-07 | Spliceosome | 1.71E-09 |
| Lysosome | 8.23E-07 | Carbon metabolism | 1.18E-06 |
| JAK-STAT signaling pathway | 9.12E-07 | Glycolysis / Gluconeogenesis | 1.39E-06 |
| Kaposi sarcoma-associated herpesvirus infection | 2.61E-06 | DNA replication | 1.48E-06 |
| Chemokine signaling pathway | 3.58E-06 | Biosynthesis of amino acids | 7.19E-06 |
| Hepatitis B | 4.45E-06 | RNA degradation | 6.75E-05 |
| Measles | 8.77E-06 | Ribosome biogenesis in eukaryotes | 9.44E-05 |

| **Top 10 KEGG pathways downregulated in MED7-depleted HeLa cells treated with VE822** | **p-value (n=3)** | **Top 10 KEGG pathways downregulated in MED7-depleted HeLa cells treated with VE822** | **p-value (n=3)** |
| --- | --- | --- | --- |
| Regulation of actin cytoskeleton | 4.41E-08 | Glycolysis / Gluconeogenesis | 7.22E-05 |
| Bacterial invasion of epithelial cells | 2.44E-07 | Mineral absorption | 0.00181668 |
| Endocytosis | 2.75E-07 | Cysteine and methionine metabolism | 0.00239363 |
| Focal adhesion | 1.74E-06 | Wnt signaling pathway | 0.00307454 |
| Viral carcinogenesis | 4.01E-06 | Ribosome | 0.00338645 |
| Tight junction | 5.75E-06 | Nitrogen metabolism | 0.00476657 |
| ErbB signaling pathway | 5.51E-05 | Insulin resistance | 0.00545707 |
| Human immunodeficiency virus 1 infection | 5.51E-05 | MicroRNAs in cancer | 0.00699461 |
| Human cytomegalovirus infection | 9.10E-05 | Signaling pathways regulating pluripotency of stem cells | 0.00746839 |
| Fc gamma R-mediated phagocytosis | 0.00011321 | Metabolic pathways | 0.00867105 |

*2. The authors demonstrate that loss of either MED12 or LIAS appears to stabilize replication fork (RF). But this is just an observation without any mechanistic insight as to how the RF stabilization is achieved. Appreciably, the LIAS function in this process might be very difficult to dissect, however, there are some obvious avenues to explore for MED12. Given that MED12 is part of a large complex involved in transcription regulation, it would make sense to test 1) if loss of other subunits of the Mediator complex would have the same effect on RF stability – even if they didn’t score as high as MED12 in the screen; 2) if the cross-talk between transcription and replication is important for the observed effect.*

We thank the reviewer for this useful comment. In the revised manuscript, we show that the role of MED12 in ATRi resistance is distinct from the Mediator complex, and from transcription in general. As we describe in the revised manuscript, MED12 is a member of the CDK8 regulatory module of the Mediator complex which is only transiently associated with the core module of the mediator complex. We show now that unlike MED12 knockdown, depletion of core Mediator subunits, or of other CDK8 module subunits, does not result in ATRi resistance or increased fork speed in ATRi-treated cells (**new Fig 6C-G, S6A-E).** Moreover, inhibition of RNA polymerase II activity by α-amanitin treatment does not affect ATRi sensitivity (**new Fig S5**). Another possibility we explored is a potential involvement of R-loops in MED12-mediated ATRi sensitization, since R-loops formed during transcription can act as replication barriers and are potentially toxic to ATR-inhibited cells. However, depletion of the R-loop disassembly factor RNASEH2, which was previously found to result in ATRi sensitivity (PMID: 30532030), increased the ATRi sensitivity of MED12-deficient cells and reduced fork speed in these cells upon ATRi treatment (**new Fig 6A-B, S4**). This argues that R-loops are still present in MED12-deficient cells, and thus the ATRi resistance of these cells cannot be explained by reduced R-loop formation.

These findings indicated that the regulation of ATRi sensitivity is a separate function of MED12 distinct from the Mediator complex. Interestingly, a previous report identified MED12 in an RNAi screen for regulators of sensitivity to inhibition of ALK and EGF receptor tyrosine kinases (PMID: 23178117). Similar to the ATRi case we investigate here, this role was found to be specific to MED12 and not involving other Mediator subunits. Instead, the phenotype was mechanistically linked to TGFβ pathway activation, as MED12 was found to suppress TGFβ Receptor 2 maturation resulting in its reduced expression on the cell surface. In the revised manuscript, we show that ATRi resistance by MED12 depletion also involves hyper-activation of the TGFβ pathway through increased expression of the TGFβ Receptor 2 (**new Fig 7A-C, S6E, S7**). Importantly, we moreover show that the increased fork speed in the presence of ATRi observed in MED12-depleted cells is also dependent on this TGFβ pathway hyperactivation (**new Fig 7D**). Thus, our results indicate that MED12 suppression of the TGFβ pathway results in ATRi sensitivity, and identify a novel role for the TGFβ pathway in replication fork stability mediated by ATR.

*3. To be able to draw a firm conclusion that CRISPR activation screen has actually worked, the authors would have to validate more than one hit per cell line.*

In the revised manuscript, we included analyses of two additional hits, namely MYCT1 (hit in HeLa cells) and MGAT4A (hit in MCF10A cells) in the **new Fig 9C, S9C-D.**

**Reviewer #3**

We are glad that the reviewer found that “*the aim of the study is important and clinically significant.”.* We thank the reviewer for their helpful comments. We have addressed these comments in the revised manuscript as indicated below:

*The purpose of including data from both, knockout and activation screens is unclear, especially because the activation library screen validation is very superficial. The activation screen does not necessarily complement or strengthen the findings of the knockout screen. It may be better to describe only on the knockout screen*

As detailed below in our response to the reviewer’s comment #7, in the revised manuscript we present additional validation of the activation screen (**new Fig 9C, S9C-D**). We include analyses of two additional hits, namely MYCT1 (hit in HeLa cells) and MGAT4A (hit in MCF10A cells). In line with the reduced overlap between the two cell lines observed in our screens, and with our previous findings with RPL35A and IRF2BP2, we found that MYCT overexpression, but not MGA4A overexpression, promotes ATRi resistance in HeLa cells.

It is true that we have not mechanistically pursued the hits from the activation screen. However, while such mechanistic studies are beyond the scope of this manuscript, we do believe that it is important to publish the result of the activation screens. These results are informative to the field and we believe will stimulate further research. Unlike knockout screens, activation screens are still far from mainstream, and the technology is in its infancy. More importantly, there is an evolving understanding of the interpretation of activation screens, which unlike knockout approaches do not involve on/off switches but rather fine modulation of genetic pathways. Publishing our integrative approach and datasets, with their inherent strengths and limitations, may prove useful to other researchers in both the genome stability field, as well as to those in other fields pursuing functional genomic approaches.

*It may be better to describe only on the knockout screen and focus on one pathway (e.g. replication or apoptosis) to provide a mechanistic insight into ATRi resistance. At present, it seems to be like a list of pathways and genes involved in ATRi resistance without any clear understanding.*

As mentioned above in the response to the comments #1 and #2 of Reviewer 2, in the revised manuscript, we present significant mechanistic insights for both MED12 (replication fork protection) and LUC7L3 (regulation of apoptosis). In particular, we show that the role of MED12 in ATRi sensitivity is independent of the Mediator complex or of transcription in general, since depletion of other Mediator subunits such as MED7, MED13, CDK8, or CCNC does not result in ATRi resistance or increased fork speed in ATRi-treated cells (**new Fig 6C-G, S6A-E).** Moreover, inhibition of RNA polymerase II activity by α-amanitin treatment does not affect ATRi sensitivity (**new Fig S5**), arguing against a role for transcription in general. Instead, we show that MED12 suppresses the TGFβ signaling pathway and its loss promotes ATRi resistance by hyper-activating this pathway through increased expression of the TGFβ Receptor 2 (**new Fig 7A-C, S6E, S7**). Importantly, we moreover show that the increased fork speed in the presence of ATRi observed in MED12-depleted cells is also dependent on this TGFβ pathway hyperactivation (**new Fig 7D**). Thus, our results indicate that MED12 suppression of the TGFβ pathway results in ATRi sensitivity, and identify a novel role for the TGFβ pathway in replication fork stability mediated by ATR.

Moreover, in the revised manuscript we show that LUC7L3 mediates the ATRi response through BCL-X alternative splicing in conjunction with its partner RBM25. In fact, RBM25 scored highly in our original screens, ranking #201 in the AZD6738 screen. Indeed, in the revised manuscript, we show that RBM25 depletion results in ATR inhibitor resistance (**new Fig 4C, 4D**). Moreover, we show that similar to loss of LUC7L3, RBM25 depletion reduces the levels of pro-apoptotic BCL-X_short_ isoform upon ATRi treatment (**new Fig 4F**). Overall, these findings indicate that LUC7L3 exerts its effect on ATRi resistance through its partner RBM25 and indicate that the alternative splicing of the BCL-X apoptosis regulator by the LUC7L3-RBM25 pathway regulates the cellular resistance to ATR inhibitor treatment.

As mentioned above, we strongly believe that publishing all screens within one manuscript will benefit the research community, both by providing access to a rich gene-level data resource for ATRi response, but also by allowing the critical evaluation of the strength and limitations of such integrative genome-wide genetic approaches.

*Other concerns and suggestions to strengthen the manuscript are listed below.
1) The authors should incorporate the dataset from published ATR inhibitor CRISPR knockout screens e.g. Hustedt et al, 2020 (*[*https://royalsocietypublishing.org/doi/10.1098/rsob.190156*](https://urldefense.com/v3/__https:/royalsocietypublishing.org/doi/10.1098/rsob.190156__;!!Ls64Rlj6!jcRe3BetJQ1JFIvFyylgfV7nU7lyRkPa4yFhVro3Y7e8GIEI_OX4i9JLeUi4ZOjq1Ak9$)*). This reference should be included in the paper. This will represent a conserved set of genes and should improve the overall confidence of identifying genes for downstream analysis.*

We thank the reviewer for pointing out this omission. We are now citing this publication in the revised manuscript. We cannot incorporate the datasets from this publication in our analyses, as these screens were performed under different experimental conditions than ours since they were screening for gene knockouts driving ATRi sensitivity, rather than resistance as in our manuscript.

*2) Fig 2A, what was the reason for selecting top 500 genes for the Venn Diagram? A cut-off to include genes in the Venn Diagram should be based on their functional significance or logP value. Including genes that do not have a significant effect is not meaningful.*

We now re-analyzed the data by using a cut-off based on logP value (namely logP lower than -2.0). In the revised manuscript, we present these analyses for all screens performed (**new Fig 2A-D, 8A-C, Tables S2 and S4**).

*3) Fig 2B: The algorithm used to calculate random probability of finding common hits should be clearly explained.*

In the revised manuscript, we explain how the random probability was calculated in the Statistical Analyses paragraph of the Methods section.

*4) Fig 3D-F. The conclusions drawn from the data shown in these figures is not convincing. Clonogenic survival assay results shown in Fig 3C are clear and convincing. Similar assay should be performed for validation of the seven top candidates in other cell lines.*

In the revised manuscript, we show that knockdown of six out of the seven hits investigated resulted in ATRi resistance in clonogenic assays in 8988T cells (**new Fig 3G**). Unfortunately, MCF10A cells do not form countable colonies, thus clonogenic assays are impracticable in these cells.

*5) Fig 4B: ATR inhibitors induce DNA damage. Knockdown of 7 genes have been shown to reduce fraction of apoptotic cells, the authors should comment on DNA damage status by performing gamma-H2AX/53BP1 staining after knockdown.*

In the revised manuscript, we show that γH2AX levels are not obviously altered by depletion of the top hits (**new Fig S2C**).

*6) Fig 4: RT- PCR showing a decrease in BCL-X short mRNA in response to LUC7K3 knockdown should include BCL-X long form as a control. What does set 1 and set 2 mean? If they represent two different experimental sets, then why only one GAPDH control?*

The RT-PCR for BCL-X_long_ is more difficult to interpret as the signal we observe is very strong, making difficult to distinguish changes in band intensity. However, in the revised manuscript we show by western blot that the levels of BCL-X_long_ protein increase upon knockdown of LUC7L3 or RBM25 (**new Fig S3C**), corresponding to the observed decrease in the short form.

We apologize for not clearly indicating this in the original submission: set 1 and 2 are two different primer sets, amplifying the same region. This is why they have the same GAPDH control. In the revised manuscript, we clearly labeled them in the figure as primer sets.  *7) Fig 6: The activator screen is very preliminary with not very convincing validation data (results shown in Fig 7E are not convincing) or mechanistic insights. Why only two genes identified in activation screen in HeLa cells were validated but none in MCF10A cells screening. The argument to explain the lack of overlap of genes identified in the two cell lines that ATRi resistance will be different in Hela and MCF10A is contradicted by a conserved response that was observed in the knockout screen genes.*

In the revised manuscript, we present additional validation of the activation screen (**new Fig 9C, S9C-D**). We include analyses of two additional hits, namely MYCT1 (hit in HeLa cells) and MGAT4A (hit in MCF10A cells). In line with the reduced overlap between the two cell lines observed in our screens, and with our previous findings with RPL35A and IRF2BP2, we found that MYCT overexpression, but not MGA4A overexpression, promotes ATRi resistance in HeLa cells. Unfortunately, despite our best efforts, we were not able to obtain stable overexpression lines in MCF10A cells, even though we used the same constructs that were successful in HeLa cells. Due to this technical challenge, we were unable to validate the results in MCF10A cells. Nevertheless, our screen results and their validation in HeLa cells are in line with the idea that gain of function-based (gene overexpression) mechanisms of ATRi-resistance are different between the two lines.

We agree with the reviewer that the conservation between HeLa and MCF10A hits appears to be different in the knockout compared to the overexpression approaches. We would like to point out that we only performed the knockout screens in HeLa cells, so we do not know if the pattern of ATRi resistance is conserved at the level of the whole gene library. But it is true that we validated the 7 top common hits in multiple cell lines, including MCF10A, and showed that they have the same phenotype as in HeLa cells -namely ATRi resistance. While we do not understand yet the significance of the differences observed between the overexpression and knockout screens in terms of conservation of gene hits between HeLa and MCF10A cells, we speculate it may reflect differences between loss of function (knockout) *vs* enhanced function (overexpression) approaches. Gene knockouts represent an on/off switch which will inactivate biological pathways, resulting in a phenotype which is less likely to depend on the cell type or transcriptional program of the cell. In contrast, gene overexpression will enhance a biological pathway which in many cases is already active in the cells. Ultimately, the phenotype may depend upon a complex interaction with other pathways and processes operating in the respective cells, which is affected to a much larger extent by the biology and transcriptional program of each cell type. Thus, knockout screens may be more prone to identify common mechanisms among cell lines compared to overexpression screens. We now discuss this more thoughtfully in the Discussion section of the revised manuscript. In any case, we believe it is important to report our data, even though the results are perhaps surprising, in order to encourage further research.

*8) Knockdown results of only four genes are shown in Suppl. Fig 1. If good antibodies are not available, qRT-PCR should be performed. Showing effect on ATRi resistance without the knockdown data is not very helpful.*

We now added, in the **new Fig S1**, western blots showing the knockdown for all 7 genes validated, including RETSAT, KNTC1, and LIAS -which were missing in the original submission. We also include blots showing the knockdowns for all the other genes we investigated for the mechanistic experiments included in the revision, including RBM25, RNASEH2, MED7, MED13, CDK8, and CCNC (**new Fig S3B, S4, S6A-D**)

*9) Suppl. Fig. 2A. The GAPDH Western blot for siLIAS should be replaced with a better control blot.*

We replaced the blot with a different exposure, clearly showing GAPDH expression.
