# Supplementary material for: Dual genome-wide CRISPR knockout and CRISPR activation screens identify mechanisms that regulate the resistance to multiple ATR inhibitors
Source: PLoS Genet. 2020 Nov 2;16(11):e1009176. doi: 10.1371/journal.pgen.1009176 (PMC7660927; doi:10.1371/journal.pgen.1009176)
Supplement: Attachment — Submitted filename: Response to referees.docx [file pgen.1009176.s017.docx]

**Response to referees**

We would like to thank the reviewers for their comments on our revised manuscript.

**Reviewers #1 and #3**

We are happy to learn that these reviewers found that our revised manuscript satisfactorily addressed their concerns.

**Reviewers #2**

We are glad that the reviewer found that our revised manuscript “is significantly improved.” Below, please find our point-by-point reply to reviewer’s remaining comments (our responses in red font)

1. In my view, including activation screens into this manuscript does not add much value to it. Even though the authors have validated a couple of additional hits in viability assays, the lack of mechanistic insight into why overexpression of those proteins modulates cellular responses to ATR inhibition, remains to be elucidated. Thus, it would make sense to leave the activation screens out and just focus on the knock-out screens. That said, in Figure 1C, the last two diagrams depicting activation screens in HeLa and MCF10A are identical and, in the future, should be contracted into one.

While we do understand the reviewer’s point, we would like to respectfully argue that the activation screens report datasets which are likely to be useful to the field. Thus, we do believe that it adds value to our manuscript. Leaving it out of the manuscript simply because we are not pursuing mechanistic experiments for hits from this screen does not seem to us like the appropriate approach, since we do not see any benefit from doing this. Instead, we believe that we would be doing a disservice to the field by not publishing these datasets. Thus, we would like to kindly request to keep this screen in the manuscript. In the revised manuscript, we updated Figure 1C by contracting both activation screens into the same depiction, as requested by the reviewer.

2. Regarding the mechanism of ATRi resistance caused by loss of MED12, the authors’ interpretation of the data in Fig. 6A&B, appears to be dubious. The claim that RNASEH2 depletion significantly reduces ATRi resistance (Fig. 6A) and RF speed (Fig. 6B) in MED12-depleted cells is hard to accept, given how minor the differences are. These data do not rule out that MED12 loss suppresses R-loop formation. In fact, it could be interpreted in a way that since loss of MED12 might suppress R-loop formation, the concomitant loss of RNASEH2 has only a minor effect on RF speed and viability in the presence of ATRi in MED12-depleted cells. As MED12 is not the only contributor to R-loop formation/resolution, in its absence, some R-loops can still form; hence, the residual effect of siRNASEH2 in MED12-depleted cells. To unequivocally the issue, the authors would have to experimentally test for abundance of RNA:DNA hybrids in their various genetic backgrounds. If those experiments are not conducted, however, I suggest that the authors at least modify their interpretation of the data in order not to exclude a very plausible hypothesis.

While the differences observed are statistically significant and are comparable to the impact of RNASEH2 depletion in wildtype cells, we do agree with the reviewer that we cannot exclude the possibility that R-loops do play a role in MED12-mediateed ATRi sensitivity. In the revised manuscript, we address this possibility in the Discussion section (page 16) as indicated by the reviewer.

3. Point to add to discussion. The authors have tested the additional components of the Mediator complex in HeLa cells and their data show convincingly that MED12 has a separate function in mediating ATRi responses. Given that the Mediator proteins such as MED13, CDK8, CCNC have previously come up in ATRi screens together with MED12 (e.g. Wang et al., 2019, Oncogene), is it possible that the effects seen here are cell line specific to HeLa cells?

We agree with the reviewer that, while our studies clearly show a Mediator complex-independent role of MED12 in regulating ATRi resistance, we cannot rule out that the Mediator complex itself does impact the cellular response to ATRi, and that this impact may be cell type specific. We now comment on this in the Discussion section of the revised manuscript (page 16).
